# Cost comparison of asthma treatments in 12-week study: caution about matching and short observational follow-up

**DOI:** 10.1186/s40248-016-0076-x

**Published:** 2016-11-02

**Authors:** David B. Price, Job F. M. van Boven, Lisa M. Law, Alessandra Cifra, R. Brett McQueen

**Affiliations:** 1 Academic Primary Care, Division of Applied Health Sciences, Polwarth building, University of Aberdeen, Foresterhill, Aberdeen, AB25 2ZD UK; 2 Department of General Practice, Groningen Research Institute for Asthma and COPD (GRIAC), University Medical Center Groningen, University of Groningen, Antonius Deusinglaan 1, 9713 AV Groningen, The Netherlands; 3 Research in Real Life, 5 Coles Lane, Oakington, Cambridge, CB24 3BA UK

**Keywords:** Matched cohort, Observational, Asthma outcomes

## Abstract

In the absence of randomisation, observational studies must take extra care to create treatment groups that are comparable in terms of key characteristics. Various matching methods exist which can create sound comparisons, minimising confounding where possible. A recent observational study by Dal Negro et al. carried out a cost analysis comparing two asthma medications. They report strong conclusions which favour one treatment over the other, however they include little discussion on the limitations of their study. The purpose of this letter is to comment on the weaknesses of the study design, including the level of matching used, and to urge readers to consider these issues alongside the interpretation of results.

## Dear editors,

*Multidisciplinary Respiratory Medicine* recently published the following study about asthma treatments: *“Fluticasone furoate/Vilanterol 92/22 μg once-a-day vs Beclomethasone dipropionate/ Formoterol 100/6 μg b.i.d.: a 12-week cost analysis in mild-to-moderate asthma”*, by Dal Negro et al. We are moved to comment on this research as there are several issues that we believe should be brought to the attention of your readers to allow for a more balanced interpretation of its results.

The first issue is that there is potentially serious confounding of the results due to poor study design. In an observational study where no randomisation is carried out, the authors are right to have applied propensity-score matching, and have matched patients using age, sex, FEV_1_ and co-morbidities. However, in a study where asthma outcomes are being compared, it is vital that asthma severity and treatment history are used as matching criteria. FEV_1_ alone is not sufficient. In this study scenario, it is absolutely key to know how these patients have been treated in the baseline period. We know that they were on a combination of long-acting beta agonist and inhaled corticosteroids (LABA/ICS) for at least 12 weeks during follow-up, but the authors do not make clear when they were stepped-up to this therapy. The potential for confounding is clear, as the outcomes of a patient whose step-up was only recently initiated may be different to those of a patient who has had a longer period of treatment stability. It is also key to know the event history (including exacerbations and hospitalisations) of the study sample. These factors are strongly predictive of future exacerbations [1] and the results of a study which fails to account for these may not be reliable. The reliability is questioned further due to the small sample size of the study (only 40 patients in each group), in which an imbalance between treatment groups could be more problematic. The follow-up period of 12 weeks is far too short to assess asthma outcomes; longer-term observation periods (at least 6–12 months) are needed to take into account variability in outcome (especially given that asthma is a chronic disease) and confounders such as seasonal variability. The authors acknowledge this limitation, but mainly in order to present it as the sole reason for a lack of significance in the difference between hospitalisation costs.

The second issue concerns the relevance and interpretation of the results of the cost analysis. The authors carried out a cost-consequence analysis, where disaggregated changes in clinical and cost outcomes are estimated. Reporting results in a disaggregated way shifts the burden of interpretation on to the consumer of the research or clinical decision-maker [2]. What would be more useful is to present the change in costs relative to change in consequences, i.e. cost-effectiveness analysis. As for the results, the mean reductions in costs for GP visits, specialist visits, and hospitalisations are small and, whilst statistically significant for GP and specialist visits, show very small shifts in costs. For example, as reported by the authors, the cost of an asthma-related hospitalisation with a comorbid disease was 2537 Euros and 1832 Euros without a comorbid disease. The change in cost of hospitalisation (173 Euros) between the groups was not statistically significant and it could hardly be considered a meaningful change for health care providers, as it accounts for roughly 7–9 % of the cost of one hospitalisation. Similarly, costs for GP and specialist visits may have been statistically significant, but lack meaningful changes in costs that would result in more efficient or affordable use of one treatment over another in this analysis. Based on these results, it is misleading to claim in the conclusions that fluticasone fuorate/vilanterol “showed clear potential for enhanced clinical outcomes and reduced costs”. A more likely conclusion would carefully claim that no meaningful changes in costs occurred at 12 weeks and that extended follow-up with larger sample sizes are needed prior to making claims on cost reductions.

There are some items in the paper that deserved further discussion from Dal Negro et al. The first is the relevance of their dose comparison. Although dose choices are largely at the discretion of clinicians, based on the Summary of Product Characteristics (SPC) of fluticasone fuorate/vilanterol and clinical trial data comparing fluticasone fuorate and fluticasone propionate, [3–5] the dose of beclomethasone dipropionate equipotent to fluticasone fuorate should be higher than the dose used in this study. It would be of interest to hear the authors’ perspective on the usefulness of their comparison. The second item is that the rate of observed hospitalisations in this mild to moderate study sample is high in comparison with the literature. In other studies, hospitalisation rates have been up to ten times less over the equivalent follow-up period, even in patients with uncontrolled asthma [6, 7]. This deserves discussion as it raises the question of the representativeness of this sample. Thirdly, the method of adherence measurement is not made clear in the paper. If we assume dose counters on the devices were utilised, there is an issue of comparability as the Nexthaler’s adherence monitor counts inhaled doses, whereas the Ellipta counts loaded doses. It is a relevant matter to clarify as the authors write that they have used adherence data to compute the cost of medications.

Lastly, an important limitation of the study is that emergency department visits were not included in the cost analysis, though this event is considered one of the core recommended cost outcomes for asthma [8].

The points discussed above should be carefully considered when interpreting the results of this paper. We hope that they will assist readers in making a fairer assessment of Dal Negro’s study.

## References

[1] Price D, Wilson AM, Chisholm A, Rigazio A, Burden A, Thomas M (2016). Predicting frequent asthma exacerbations using blood eosinophil count and other patient data routinely available in clinical practice. J Asthma Allergy.

[2] Gray AM CP, Wolstenholme JL, Wordsworth S. Applied Methods of Cost-Effectiveness Analysis in Health Care. New York: Oxford University Press; 2011.

[3] Relvar Ellipta 92 micrograms/22 micrograms inhalation powder, pre-dispensed. http://www.medicines.org.uk/emc/medicine/28496. Accessed 4 July 2016.

[4] Lotvall J, Bleecker ER, Busse WW, O’Byrne PM, Woodcock A, Kerwin EM (2014). Efficacy and safety of fluticasone furoate 100 ug once-daily in patients with persistent asthma: a 24-week placebo and active-controlled randomised trial. Respir Med.

[5] Woodcock A, Bleecker ER, Lotvall J, O’Byrne PM, Bateman ED, Medley H (2013). Efficacy and safety of fluticasone furoate/vilanterol compared with fluticasone propionate/salmeterol combination in adult and adolescent patients with persistent asthma: a randomized trial. Chest.

[6] Chong J, Haran C, Chauhan BF, Asher I (2015). Intermittent inhaled corticosteroid therapy versus placebo for persistent asthma in children and adults. Cochrane Database Syst Rev.

[7] Edwards SJ, von Maltzahn R, Naya IP, Harrison T (2010). Budesonide/formoterol for maintenance and reliever therapy of asthma: a meta analysis of randomised controlled trials. Int J Clin Pract.

[8] Akinbami LJ, Sullivan SD, Campbell JD, Grundmeier RW, Hartert TV, Lee TA (2012). Asthma outcomes: healthcare utilization and costs. J Allergy Clin Immunol.

