# Supplementary material for: Cost comparison of asthma treatments in 12-week study: caution about matching and short observational follow-up
Source: Multidiscip Respir Med. 2016 Nov 2;11:39. doi: 10.1186/s40248-016-0076-x (PMC5090892; doi:10.1186/s40248-016-0076-x)
Supplement: Related texts

# Reply: Authors’ response

**Authors:** Roberto W. Dal Negro, Sergio Iannazzo

We read carefully the letter received, and concerning some criticisms to the paper “Fluticasone furoate/Vilanterol 92/22 μg once-a-day vs Beclomethasone dipropionate/ Formoterol 100/6 μg b.i.d.: a 12-week cost analysis in mild-to-moderate asthma*”*, recently published in *Multidisciplinary Respiratory Medicine (*2016;11:20).

We are rather surprised by the unfair and unbalanced description of our study. The Authors of the letter essentially mention that we draw “strong conclusions which favour one treatment over the other” based on a poor study design. We do not really see the strong conclusions in our paper, when the final sentence in the published abstract stands out literarily as: “Even if both ICS/LABA combinations were checked over a limited period of time, they seem characterized by a different profile in terms of their effect on lung function and of their economic impact on mild-to-moderate asthma. The once-daily inhalation of combined Fluticasone furoate/Vilanterol 92/22 μg showed the potential for enhanced clinical outcomes and reduced costs when compared to Beclomethasone dipropionate/Formoterol 100/6 μg b.i.d.” In other terms we report a trend, which is in the numbers, but that has to be confirmed by a longer observation.

Furthermore, we wish to pinpoint that the study is an observational, retrospective study, and that data were obtained from an institutional general database which is collecting files of asthma patients managed in real-life. In other words, as clearly perceived from the paper, the study does not belong to the traditional pharmacological trials where subjects have to be preliminarily randomized into different arms. Actually, this was the main reason for the adoption of the propensity matching score. The Authors of the letter believe that we correctly applied the method but left key information unmanaged, leading to a poor design. Essentially, they mention asthma severity and treatment history (i.e. when patients were stepped up to study therapies) as key characteristics that should have been used as matching criteria. We believe this criticism derives from a misunderstanding of our study. Differently from the opinion of the Authors’ letter, we do not perceive any “serious confounding”, because it appears very clearly that not-severe asthma was the unique target of the survey. Actually, the high mean basal FEV_1_ value (>82 % pred.), together with the low clinical impact already registered in basal conditions do represent clear real-life indices from this point of view, independently of previous patients’ therapeutic strategies.

Moreover, all automatically selected patients were equally stepped-up exactly at their recruitment date (such as 12 weeks ± 1 week before the end of the survey): previously, they were regularly treated and were in consolidated stable conditions before their step-up date. They also were well matched at the step-up date and absolutely undistinguishable, both in terms of their lung function and of clinical impact. To underline that basal data were obviously referred to the period immediately preceding the start of the survey, and then what the Authors of the letter were requiring and are worried of was already reported in the paper (Table two, column A for both the patients’ samples).

As concerning the duration of the survey, we only wish to recall that, differently than in COPD, several clinical trials carried out in asthma with the aim to check and compare the effects of different drugs (vs placebo or not) had, and still have, a 12-week duration, and that also international guidelines were affected by some papers of this duration.

As concerning the extent of the clinical impact reported at the step-up, we can only comment that it should be unbelievable if the original exacerbation and the hospitalization rates would have been higher in not-severe asthma patients, already treated with other therapies, as the patients of the study were.

Consequently, the achieved economic outcomes may appear not impressive at glance. Nevertheless, the significant difference registered in n. visits between the treatments should not be neglected because occurring over a 12-week period only, and this particular piece of evidence was still missing in literature, to our knowledge, when comparing these two drugs. Obviously, nobody would have expected highly dramatic changes in hospital admission and n. exacerbation rates in two sample of patients of this severity, who were stepped up for three months only. If occurring, it would have been regarded as a biased outcome, in our opinion.

The only simple message from the present paper is that some significant and unexpected differences in clinical outcomes are occurring when comparing the two treatments, even if within only 12 weeks, and in two small samples of subjects. The differences registered (such as in lung function and in reduction of unscheduled GPs’ and specialists’ visits) are in fact reflecting two interesting aspects of asthma governance which proved in favour of Fluticasone furoate/Vilanterol 92/22 μg once-a-day) rather than of Beclomethasone dipropionate/Formoterol 100/6 μg b.i.d., stepped up for a same period of time. Some suggestions for explaining this difference had been reported in the paper. Once again, this small piece of evidence was not available yet.

The authors of the letter also complain about the relevance of the results of a cost analysis, or better that our cost-analysis left disaggregated changes in cost and clinical outcomes for the decision-makers, instead of cooking for them a full cost-effectiveness analysis. This seems in contradiction with their other criticism of poor study design. We all well know that the cost-effectiveness is the most informative analysis among the health economics armamentarium, however we felt that the results of our study were just preliminary and as a consequence their use in a cost-effectiveness analysis would have been inappropriate. This again should reassure the reader that we are not drawing “strong conclusions” on our data.

Finally, just to reassure the Authors of the letter, we wish to inform that the survey had been originally planned for a 12-month duration, and that results reported in the present, recently published paper only represent the very first cluster of data. The overall results collected over one year are in advanced calculation, and their submission has been already planned for the next fall.
